# Risperidone-induced Somnambulism: A Case Report and Brief Review of Literature

**DOI:** 10.7759/cureus.7238

**Published:** 2020-03-10

**Authors:** Ahmad Najmi, Faisal Siddiqui, Avik Ray, Ratinder Jhaj, Balakrishnan Sadasivam

**Affiliations:** 1 Department of Pharmacology, All India Institute of Medical Sciences Bhopal, Bhopal, IND; 2 Department of Psychiatry, All India Institute of Medical Sciences Bhopal, Bhopal, IND

**Keywords:** schizophrenia, risperidone, somnambulism, case report, adverse drug reactions

## Abstract

Atypical antipsychotic drugs have been recommended as the first-line agents in the treatment of schizophrenia. They are usually not associated with extrapyramidal symptoms and hyperprolactinemia. There is no previously reported case on somnambulism associated with risperidone intake. We report a case of risperidone-induced somnambulism in a schizophrenic patient. Somnambulism occurred while the patient was on a dose of 3 mg of risperidone per day. The dose was reduced to 2 mg per day along with the addition of clonazepam 0.5 mg daily at night. No further episode of somnambulism was reported after this. This case suggests the association of risperidone with occurrence of somnambulism. Further evidence to strengthen the causality of this needs to be gathered. This calls for prompt pharmacovigilance reporting of adverse drug events associated especially with antipsychotics.

## Introduction

Atypical antipsychotic drugs have been recommended as the first-line agents in the treatment of schizophrenia [1]. While these agents do not entirely rule out the risks of extrapyramidal symptoms and hyperprolactinemia, the incidence is greatly reduced when administered in therapeutic ranges [2]. Somnambulism, a type of parasomnia, is characterized by a group of unwanted movements in bed during sleep or even sleepwalking. It mainly occurs in children aged four to eight years and disappears as they approach their teenage [3]. Risperidone is a potent atypical antipsychotic and acts as an antagonist/inverse agonist at serotonergic (5-HT_1A_, 5-HT_1B_, 5-HT_1D_, 5-HT_2A_, 5-HT_2C_, 5-HT_5A_, 5-HT_7_), dopaminergic (D_1-5_), adrenergic (α_1A_, α_2A-2C_) and histaminergic (H_1_, H_2_) receptors, lacking any effect on the muscarinic receptors [4]. Several studies have reported drug-induced sleepwalking in the past decade involving zolpidem, olanzapine, quetiapine, lithium and propranolol [5-8]. However, after thorough literature search, no previously reported case on somnambulism associated with risperidone administration could be found. We report a case of risperidone-induced somnambulism in a patient with schizophrenia.

## Case presentation

The patient was a 38-year-old male who had symptoms of second-order hallucinations, delusion of reference, muttering, impaired biological functions and socio-occupational dysfunction for three months. He was diagnosed with schizophrenia according to International Statistical Classification of Diseases and Related Health Problems (ICD)-10 criteria by a licensed psychiatrist and underwent treatment with an atypical antipsychotic drug, risperidone at a dose of 2 mg per day initially which was gradually titrated to 6 mg per day within three months. The patient came for monthly follow-ups. He attained remission within six months of treatment, and the dose of risperidone was reduced to 3 mg per day on which he was well maintained and the symptoms of schizophrenia did not re-appear. After one year of remission, the patient’s wife reported about a few episodes of sleepwalking with open eyes and loud talking. She mentioned that the patient had been relocating his clothes and personal belongings in his room, while he had not been able to reply to his wife. He had got back to bed and continued sleeping, while having no memory of the event the next day. He had no delusions and hallucinations, and he was relatively functional.

The patient had no personal or family history of somnambulism. He did not have any history of alcohol intake, use of nicotine or any other substance abuse. He was prescribed clonazepam 0.5 mg per night, and risperidone was continued at a reduced dose of 2 mg daily. On his next monthly visit, no episode of somnambulism was reported by his wife and no similar symptoms were reported after three months of follow-up. 

## Discussion

Somnambulism occurs during the period of slow wave sleep (stages 3 and 4) due to impairment in the normal mechanisms of arousal from sleep, leading to abnormal motor behaviors without complete awareness [9]. Atypical antipsychotics, such as olanzapine and risperidone, may enhance the slow wave sleep by blocking 5-HT_2C_ receptors, leading to various parasomnias [10]. While two cases of pavor nocturnus (night terror) due to single nightly dose of risperidone have been reported, low-dose risperidone at 2 mg daily has been used to successfully treat cases of night terrors and sleepwalking in adults [11-13]. Enhancement in the slow wave sleep due to risperidone was thought to be the reason for night terror, and the symptoms disappeared on dividing the drug dose, thus reducing its plasma concentration [11].

Sleepwalking is considered to be on the same nosologic continuum as night terrors. A longitudinal study showed that almost one-third of the children who had experienced early childhood night terrors developed sleepwalking later in their life [14]. Most of the cases of somnambulism in adults are continuation of childhood behavior triggered by a medication or psychopathology [3]. In our case, the patient did not have any personal or family history of parasomnia; this might however be due to recall bias.

Non-antipsychotics such as lithium and beta-blockers and other substances like alcohol, nicotine and opioids can also be associated with somnambulism and other sleep arousal disorders [10]. The patient did not have any history of usage of any such substances. Temporal correlation was established since the episodes of sleepwalking occurred after treatment with risperidone was started. The symptoms completely disappeared after a slight reduction of the dose of risperidone and addition of clonazepam, indicating that risperidone was the causative agent for the condition. Other precipitating factors such as fever, stress and other disorders causing sleep arousals (obstructive sleep apnea, distention of bladder, loud noise) were also ruled out [15].

Using the causality assessment algorithm of Naranjo et al., a total score of 7 was obtained, indicating a probable causative relationship between somnambulism and risperidone [16]. The case was reported to the hospital adverse drug reaction monitoring center.

## Conclusions

This is the first case of risperidone-induced somnambulism to be reported. Further evidences regarding the range of dose and plasma concentration at which risperidone is associated with somnambulism are needed. Hence, drawing attention of physicians and other health care professionals to such adverse effects associated especially with antipsychotics and prompt pharmacovigilance reporting are required.
